# Supplementary material for: VNC-Dist: A machine learning-based semi-automated pipeline for quantification of neuronal position in the C. elegans ventral nerve cord
Source: PLoS One. 2025 Aug 28;20(8):e0331188. doi: 10.1371/journal.pone.0331188 (PMC12393719; doi:10.1371/journal.pone.0331188)
Supplement: S1 Table — (DOCX) [file pone.0331188.s001.docx]

**Table S1| Primers used for CRISPR knock-ins.**

| ***unc-4(zy123[unc-4::mNG::3xFlag])*** | |
| --- | --- |
| guide | AGCGACTAATGCATTGACTAGTTTAAGAGCTATGCTGGAA |
| 5’unc4arm.F | acgttgtaaaacgacggccagtcgccggcaatttctccccgtcacgtcttc |
| 5’unc4arm.R | CATCGATGCTCCTGAGGCTCCCGATGCTCCTACACTTTTCAGTAATTCAGCAACAGTAGTCAATGCATTAGTCGCTAC |
| 3'unc4arm.F | CGTGATTACAAGGATGACGATGACAAGAGATAAatttttttaaaattcaattttgaaccgtgccc |
| 3'unc4arm.R | ggaaacagctatgaccatgttatcgatttcatttcagctctgcgagacgt |
| ***vab-7(zy137[vab-7::mNG::3xFlag])*** | |
| guide | TTAATCTGTAGAATAAGGCGGTTTAAGAGCTATGCTGGAA |
| 5'vab7arm.F | acgttgtaaaacgacggccagtcgccggcacaagtggccattactcgcaa |
| 5'vab7arm.R | CATCGATGCTCCTGAGGCTCCCGATGCTCCGTCGGTAGAATAAGGCGAGGGAGA |
| 3'vab7arm.F | CGTGATTACAAGGATGACGATGACAAGAGATAAttcactattttttggaaaaaaaaatcgg |
| 3'vab7arm.R | ggaaacagctatgaccatgttatcgatttcgttgtggcaaattgataggaa |
| ***vab-7(zy142[vab-7::mNG::T2A::mScarlet-I::H2B]*zy137)*** | |
| guide | GAGAATCTGTACTTTCAATCGTTTAAGAGCTATGCTGGAA |
| H2B_vab7c.F | ccaagtacacttccagcaagtgacgatgacaagagataattc |
| T2A_mNG.R | cctctgccctctccagatcccttgtagagctcgtccattc |
