# Supplementary figures and images for: VNC-Dist: A machine learning-based semi-automated pipeline for quantification of neuronal position in the C. elegans ventral nerve cord
Source: PLoS One. 2025 Aug 28;20(8):e0331188. doi: 10.1371/journal.pone.0331188 (PMC12393719; doi:10.1371/journal.pone.0331188)

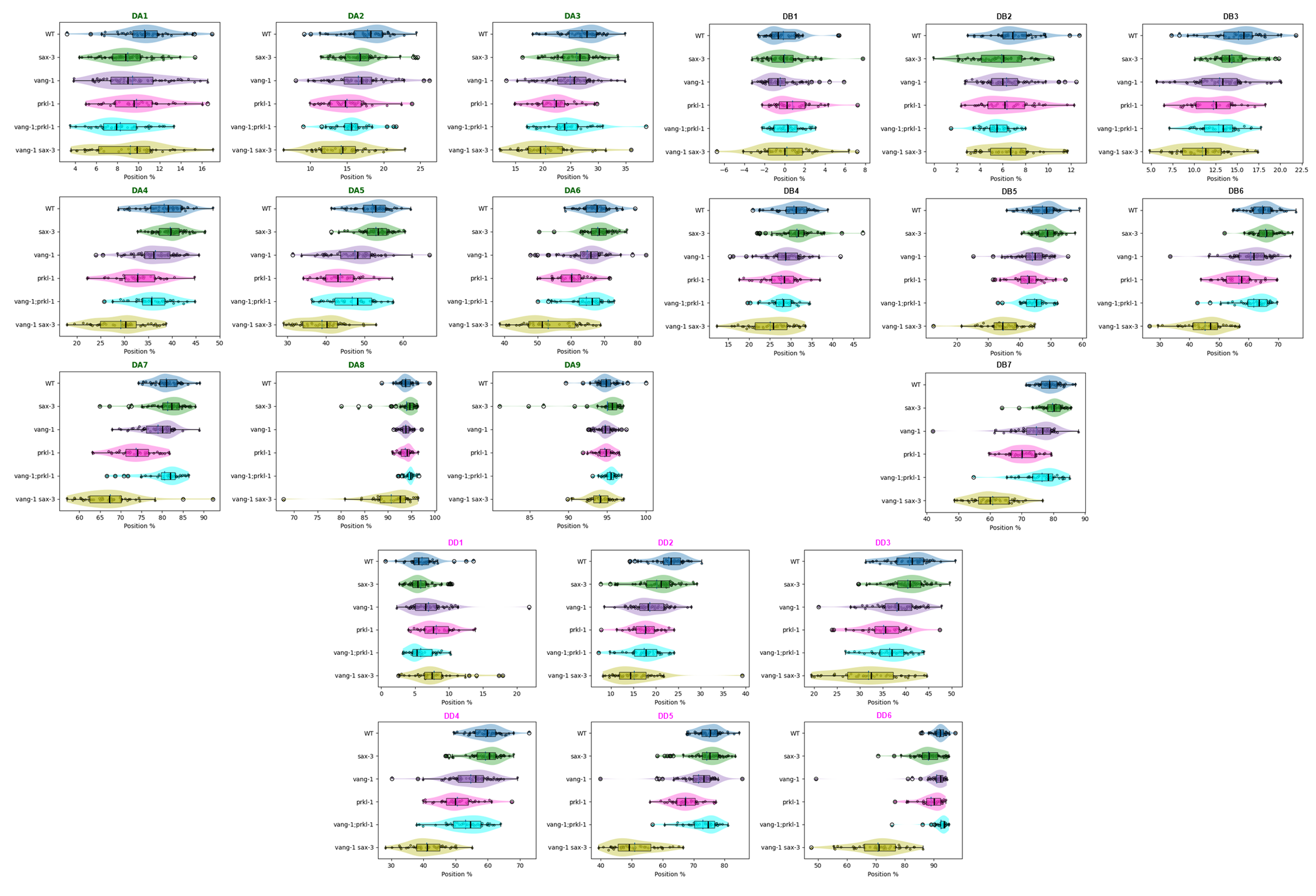

Supplement: S1 Fig — Mutant strains (prkl-1(ok3182), sax-3(zy5), vang-1(tm1422), vang-1;prkl-1, vang-1 sax-3) exhibit altered anteroposterior neuron positioning compared to WT. Distributions assess normality and highlight genotype specific effects on neuron positioning along the AP axis, with median (black) and mean (blue) indicated by vertical lines. (TIF) [file pone.0331188.s002.tif]

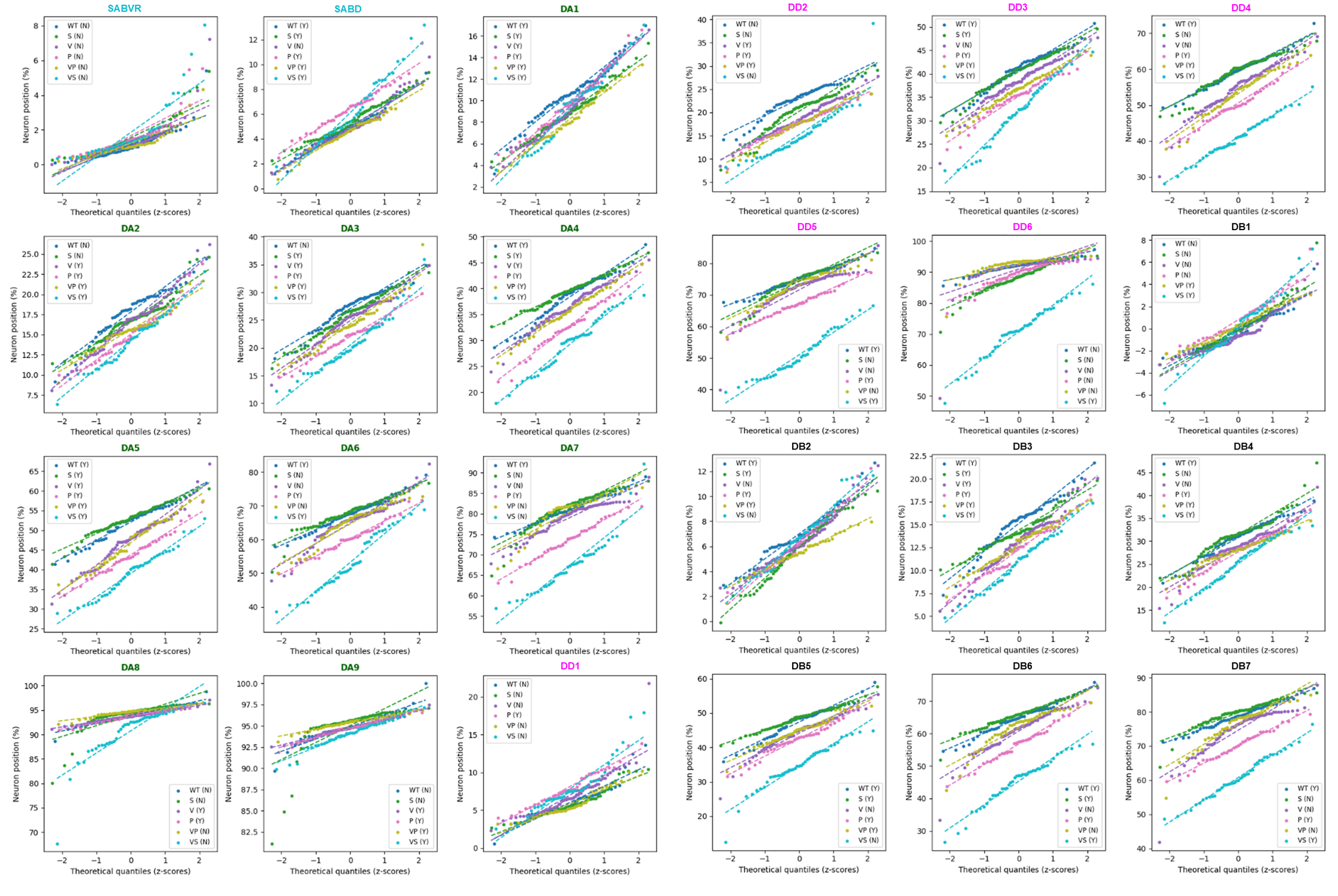

Supplement: S2 Fig — Q–Q plots with Shapiro–Wilk tests assess the normality of DA, DD and DB neuron position distributions in WT and five mutant genotypes (P: prkl-1(ok3182), V: vang-1(tm1422), S: sax-3(zy5), VP: vang-1;prkl-1, VS: vang-1 sax-3). Deviations from the diagonal indicate non-normality. Legend entries “Genotype (Y/N)” report Shapiro–Wilk test outcomes (Y = normal; N = non-normal) and are color-coded by genotype. VS (cyan) consistently has the lowest intercept (most anterior shift) across neurons, with P (pink) a close second, and VS’s markedly steeper slope reflects its larger variance. (TIF) [file pone.0331188.s003.tif]

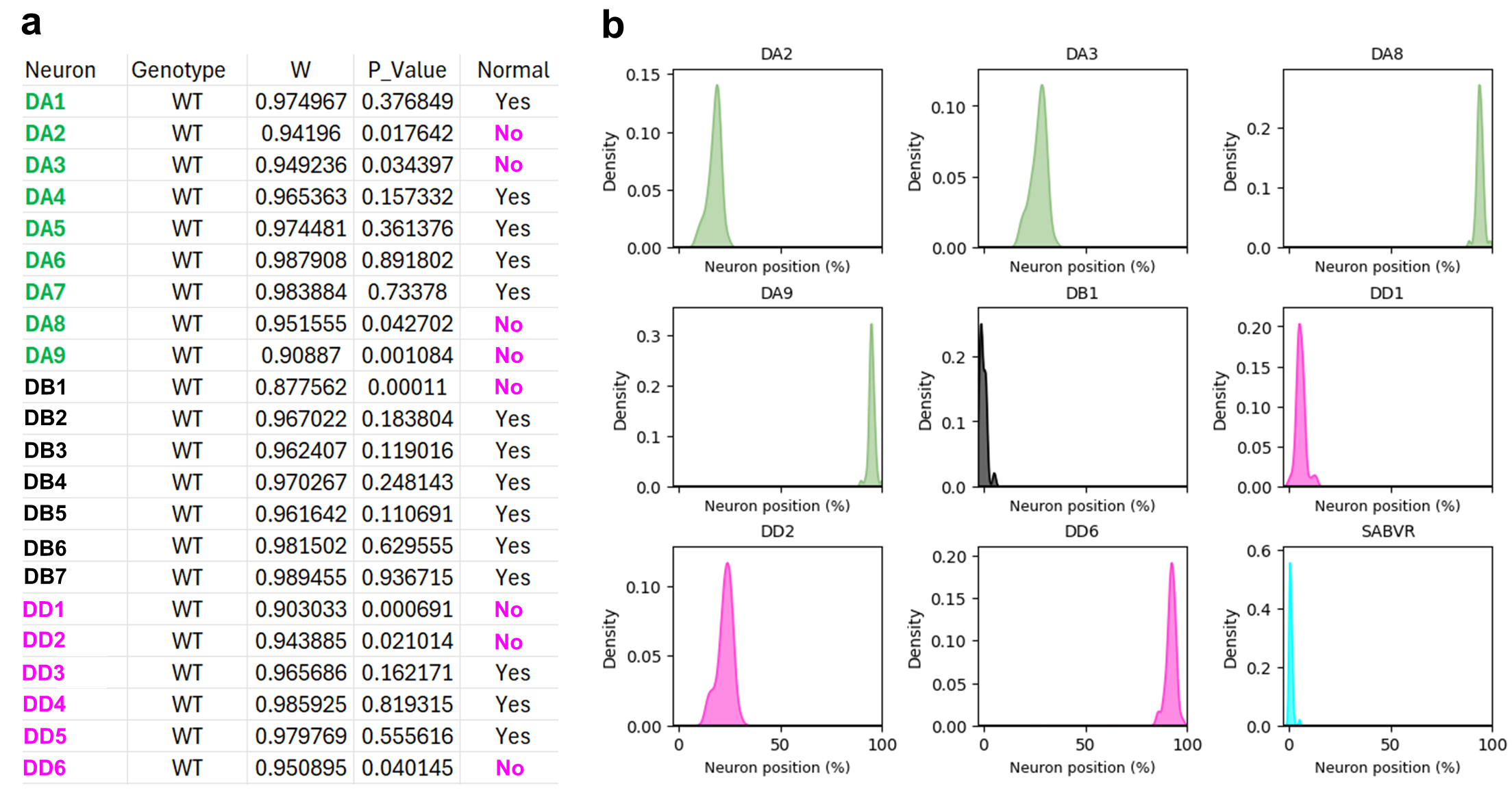

Supplement: S3 Fig — (a) Neurons with p-values > 0.05 are considered normally distributed. (b) Although DA2, DA3, DA8, DA9, DB1, DD1, DD2, and DD6 showed significant deviations from normality (p < 0.05), their Shapiro statistics, histograms, and Q–Q plots suggest they can be reasonably assumed to follow a normal distribution (n = 49). (TIF) [file pone.0331188.s004.tif]
